# Identification of EGFR as a Biomarker in Saliva and Buccal Cells from Oral Submucous Fibrosis Patients—A Baseline Study

**DOI:** 10.3390/diagnostics12081935

**Published:** 2022-08-11

**Authors:** Abirami Moorthy, Divyambika Catakapatri Venugopal, Vidyarani Shyamsundar, Yasasve Madhavan, Soundharya Ravindran, Mehanathan Kuppuloganathan, Arvind Krishnamurthy, Sathasivasubramanian Sankarapandian, Vani Ganapathy, Vijayalakshmi Ramshankar

**Affiliations:** 1 Department of Oral Medicine and Radiology, Sri Ramachandra Institute of Higher Education and Research (DU), Porur, Chennai 600116, India; 2 Centre for Oral Cancer Prevention and Research, Sree Balaji Dental College and Hospital, Pallikaranai, Chennai 600100, India; 3 Department of Preventive Oncology (Research), Cancer Institute (WIA), Adyar, Chennai 600020, India; 4 Department of Surgical Oncology, Cancer Institute (WIA), Adyar, Chennai 600020, India; 5 Department of Biochemistry, University of Madras, Chennai 600025, India

**Keywords:** OSMF, EGFR, immunohistochemistry, ELISA, biomarker

## Abstract

Oral Submucous Fibrosis (OSMF) is a chronic debilitating disease more frequently encountered in the South-East Asian population. This disease represents a public health priority as it is grouped within oral potentially malignant disorders, with malignant transformation rates of around 7–19%. Hence, early identification of high-risk OSMF patients is of the utmost importance to prevent malignant transformation. Among various biomarkers, EGFR overexpression has an unfavorable clinical outcome, poor prognosis, and low survival rates in Oral Squamous Cell Carcinoma (OSCC). The current study aimed to evaluate the expression of EGFR in saliva and exfoliated buccal cells of OSMF. Immunoexpression of EGFR was observed in healthy controls (n = 11), OSCC (n = 106), and OPMD with dysplasia (n = 56), which showed significant expression with increasing grades of dysplasia and OSCC. EGFR expression was evaluated in saliva and exfoliated buccal cells of healthy controls (n = 15), OSMF (n = 24), and OSCC (n = 10) patients using ELISA, which revealed significant expression in OSMF and OSCC. Validation studies were also performed using real-time PCR (RT-PCR) to compare gene expression in healthy controls (n = 9), OSMF (n = 9), and OSCC (n = 25), which showed significant 18-fold upregulation in OSCC and three-fold upregulation in OSMF when compared to healthy controls. Hence, saliva and exfoliated buccal cells could be considered as potential non-invasive diagnostic samples for the evaluation of high-risk patients of OSMF using EGFR as a biomarker.

## 1. Introduction

Oral cancer accounts for more than 30% of all cancers and poses a significant burden in the Indian subcontinent [1]. Tobacco and betel nut chewing habits are the main contributing factors to its increased incidence. Leukoplakia, erythroplakia, and palatal changes due to reverse smoking, which are tobacco-associated, and Oral Submucous Fibrosis (OSMF), associated with arecanut use, have been grouped within oral potentially malignant disorders (OPMD). Among the various OPMD, OSMF is highly prevalent in South-East Asia, with a high malignant transformation rate of 7–13% [2]. Early identification of high-risk OPMD using potential biomarkers can reduce the carcinomatous transformation, thereby reducing the morbidity and mortality associated with the disease [3,4]. Over recent years, biomarkers in various samples, such as serum, saliva, fresh tissue, exfoliated buccal cells, and formalin-fixed paraffin blocks (FFPE), have been studied [5,6,7]. Among the various samples used for diagnostic purposes, saliva is found to be a protein-rich fluid that is directly in contact with the oral lesions and represents an ideal source for the development of biomarkers for early detection, monitoring the progression of OPMD, and also in assessing the treatment response [4]. Numerous potential biomarkers have been evaluated in saliva and are overexpressed in OPMD and OSCC [5]. 

Epidermal Growth Factor Receptor (EGFR) is a tyrosine kinase receptor involved in various cellular activities [8]. EGFR (also known as ErbB-1/HER1) is a 170 KDs transmembrane glycoprotein belonging to the ErbB family of receptor tyrosine kinases (RTK) [9,10], involved in signal pathways associated with cancer development and progression and associated with several gene mutations [11,12]. Overexpression of EGFR has an unfavorable clinical outcome, poor prognosis, and low survival rates in Oral Squamous Cell Carcinoma (OSCC) [13,14,15]. The reliability of EGFR is proven because the molecular targets inhibiting EGFR receptors are currently under clinical trial [6]. EGFR has been found to play a role in cell invasion by induction into the Epithelial to Mesenchymal Transition [16,17]. Hence, EGFR is overexpressed in OSCC and OPMD. EGFR was found to be overexpressed in leukoplakia and oral submucous fibrosis, thus suggesting a valuable diagnostic marker for early malignancy [18]. Similar studies in Oral Submucous Fibrosis showed increased immunohistochemical expression of EGFR [19,20] and Oral Squamous Cell Carcinoma [21]. In a study done by Zanotti et al., comparing serum and salivary EGFR in oral cancer, it was found that saliva was a more reliable diagnostic and prognostic marker compared to serum [3]. However, there are contradictory results reported with salivary EGFR regarding its reliability as a diagnostic and prognostic marker in saliva [7,22].

To the best of our knowledge, the existing literature on EGFR expression in saliva and exfoliated buccal cells of OSMF and OSCC is limited. Immunoexpression of EGFR in OPMD with dysplasia compared to expression levels in different grades of OSCC will further aid in understanding the role of this biomarker in monitoring the prognosis of OPMD. Hence, the study aims to assess the expression of EGFR in saliva and exfoliated buccal cells of OSMF and OSCC patients. The current study also attempts to validate the expression of EGFR in tissue samples of OSMF and OSCC using real-time polymerase chain reaction (RT -PCR) and the immunoexpression of EGFR in OPMD with dysplasia and OSCC using IHC.

## 2. Materials and Methods

### 2.1. Patient Samples

The study was approved by the Institutional Ethical Committee (IEC No. CSP/19/MAY/77/169) and was conducted at the Department of Oral Medicine and Radiology, Sri Ramachandra Institute of Higher Education and Research, from June until August 2019. Written informed consent was obtained from all the study participants. Patient demographic details, medical history, habits, and details of clinical examination were recorded in the proforma. 

A total of n = 49 subjects were enrolled for the study and divided into Group A (n = 24), comprising Oral Submucous Fibrosis, Group B (n = 10), comprising OSCC, and Group C (n = 15), comprising healthy controls. Subjects who were less than 18 years of age, patients who were currently under treatment or previously treated for OSCC or OSMF, and those who had not given informed consent for participation were excluded from the study. Biopsy was performed in Group A and Group B patients as a routine diagnostic workup for histopathological confirmation and further treatment planning. 

### 2.2. Sample Collection

Unstimulated whole saliva was collected from Group A, B, and C by passive expectoration and spit into a 50-mL sterile tube containing proteinase inhibitor (Proteinase inhibitor cocktail (P2714, Sigma Aldrich). Patients were asked to refrain from drinking, eating, chewing tobacco, or smoking 1 h before collecting saliva. Whole saliva samples were transferred to 1.5-mL sterile microtubes and centrifuged for 3 min at 13,000 rpm. The supernatants were immediately aliquoted and stored at −80 °C within 60 min after saliva collection. The buccal exfoliated cells from the same patients were collected using a sterile buccal swab, followed by 2 min rinse with 10 mL distilled water. The samples were transferred to 1.5-mL sterile microtubes and centrifuged for 3 min at 13,000 rpm, immediately aliquoted, and stored at −80 °C [23].

In addition, formalin-fixed paraffin-embedded (FFPE) sections fom healthy controls (n = 11) and retrospective samples of OPMD with dysplasia (n = 56) and OSCC (n = 106) were obtained.

### 2.3. Measurement of EGFR Levels 

Saliva and buccal samples were thawed on ice and centrifuged at 3000 rpm at 48 °C before analysis. EGFR concentrations were determined using a commercial sandwich enzyme-linked immunosorbent assay (ELISA), according to the manufacturer’s instructions (Human EGFR ELISA kit; Wuhan Fine Biotech Co., Ltd., Hubei, China). The plate was washed twice before adding standard, sample, and control (zero) wells. 100 µL standard and sample was added to each well and incubated for 90 min at 37 °C, followed by aspiration, and further the plates were washed twice. 100 µL of Biotin-labeled antibody working solution was added to each well and incubated for 60 min at 37 °C, aspirated, and the ELISA plate was washed thrice. Additionally, 100 µL of HRP-Streptavidin Conjugate (SABC) working solution was added to each well, incubated for 30 min at 37 °C, then aspirated and the plate was washed five times. The next step was followed by the addition of 90 µL TMB substrate and incubated for 15–30 min at 37 °C. Subsequently, 50 µL stop solution was added and the absorbance was read at 450 nm immediately. Experiments were performed in duplicates and the average value was taken for the analysis. EGFR levels were determined using standard curves, reading the optical density at 450 nm on an automatic plate reader (ROBONiK; readwell TOUCH, ELISA Plate Analyser). Results were tabulated and subjected to analysis by SPSS 15 software [24].

### 2.4. Immunohistochemistry

Immunohistochemistry was performed on 4 µm sections obtained from formalin-fixed paraffin-embedded tissue (FFPE) samples. Sections were taken on slides coated with 3-aminopropyltriethoxysilane (APES). The sections were deparaffinized in xylene and rehydrated using absolute alcohol. Endogenous peroxidase activity was quenched by immersing the sections for 10 min in 0.03% hydrogen peroxide in distilled water, followed by a distilled water wash. Antigen retrieval was done with 0.05 M Tris EDTA Buffer (pH-9) in a pressure cooker for 20 min. Sections were pre-incubated with 2% bovine serum albumin (BSA) for 40 min. The sections were incubated with primary antibody EGFR (PU335: polyclonal rabbit anti-EGFR, Biogenex, CA, USA) overnight at 4 °C in 100% moisture. The BioGenex Super Sensitive^TM^ Detection System (Biogenex, CA, USA) was used to detect expression. Hematoxylin-counterstained sections were dehydrated using ascending grades of isopropyl alcohol and xylene and mounted in DPX. Known positive controls and negative controls were used. The expression of EGFR was graded and compared to the absolute normal oral mucosa. In 10 high power fields (40×), several positive cells were counted in the epithelium and connective tissue (connective tissue cells such as fibroblasts and inflammatory cells), and the % positivity was computed. Counting was done on a computer display using the software ProgRes CapturePro v2.8.8. Briefly, EGFR expression was assessed semi-quantitatively by evaluating the percentage of epithelial CT cells and, based on the expression levels of the respective proteins, they were classified as follows: (i) Mild Positive—10% to 50% EGFR expression, (ii) Intermediate Positive—60% to 90% EGFR expression, and (iii) Strong Positive—100% EGFR expression [25]. The staining intensity was measured at several levels of the epithelium (basal, stratum spinosum, and superficial). Similarly, expression in the connective tissue was also counted. Scoring for IHC was done by an oral pathologist who was blinded to the clinical details of the included patient samples.

### 2.5. Tissue Homogenization

The collected patient tissue samples (Healthy controls = 9; OSMF = 9, OSCC = 25) were sliced into small pieces using a sterile surgical blade. The tissue pieces were transferred into individual 2-mL centrifuge tubes each containing 300 µL of TRIzol reagent (Thermo Fischer Scientific Inc., Waltham, MA, USA). Two sterile beads were added to all the tubes and they were placed in a Tissue LyserLT (Qiagen Inc., Venlo, The Netherlands) for 15 min. After the lysis of tissues, 700 µL of TRIzol reagent was added to the tubes, resuspended, and transferred to fresh 1.5 mL centrifuge tubes [26].

### 2.6. RNA Isolation

First, 0.2 mL of chloroform per 1 mL of TRIzol reagent was added and the tubes were vortexed for 10 to 15 s, followed by incubation at room temperature for 3 min. The samples were centrifuged at 12,000 rpm, for 15 min at 4 °C. (The mixture was separated into a lower phenol–chloroform phase, an interphase, and a colorless upper aqueous phase. RNA remained in the aqueous phase). The aqueous phase was transferred to a fresh tube. The RNA was precipitated by mixing with isopropyl alcohol (0.5 mL of isopropyl alcohol per 1 mL of TRIzol reagent). The samples were incubated at room temperature for 10 min and centrifuged at 12,000 rpm for 10 min at 4 °C. The RNA precipitate formed a white pellet on the side and bottom of the tube. The supernatant was removed and 1 mL of 75% ethanol per 1 mL of TRIzol was added. The sample was mixed by vortexing and centrifuged at 7500 rpm for 5 min at 4 °C.

The supernatant was removed and the RNA pellet was dried briefly. The RNA pellet was not allowed to dry completely as this may greatly decrease its solubility. The RNA was dissolved in RNase-free water by flipping the tube a few times, and it was incubated for 10 min at 55 to 60 °C [27].

### 2.7. cDNA Conversion

The Quantitect Reverse Transcription Kit (Qiagen Inc.) was used for the reverse transcription of the RNA samples. A total of 2 µg of RNA was required for the cDNA conversion, which consisted of 2 major steps: elimination of genomic DNA and reverse transcription. The purified RNA sample was incubated with 2 µL of gDNA wipe-out buffer and briefly incubated at 42 °C for 2 min. The reaction mix was immediately transferred to ice. After gDNA elimination, the RNA sample was ready for reverse transcription using a master mix prepared with the Quantiscript Reverse Transcriptase, Quantiscript RT Buffer, and RT Primer mix. The entire reaction took place at 42 °C and was inactivated at 95 °C. The prepared cDNA was stored at −40 °C [28].

### 2.8. Real-Time PCR

SYBR Green-based real-time amplification was performed using the QuantiNova SYBR Green RT-PCR Kit (Qiagen Inc., Venlo, The Netherlands). A 20-μL reaction was set up, which contained 10 µL of 2× QuantiNova SYBR Green RT-PCR Master Mix, 1 µL of each forward and reverse primer (Table 1), 6 µL of nuclease-free water, and 2 μL of cDNA. The thermal profile was 30 min at 50 °C, 15 min at 95 °C, 45 cycles of 15 s at 94 °C, 30 s at Tm, 30 s at 72 °C, ending with a melting curve from 60 °C to 90 °C. All real-time amplifications were run on a Rotor Gene Q Real-Time PCR system (Qiagen). Triplicate reactions were performed for gene expression studies using EGFR, and the mean expression value was computed for the subsequent analysis. The relative expression level of the genes was calculated using the (2-ddct) method [29].

### 2.9. Statistical Analysis

Categorical variables were presented as percentages and continuous variables as mean ± standard deviation (SD). Clinical characteristics and outcomes were compared between the groups (OSMF vs. OSCC vs. healthy controls) using Dunnett’s T3 post-hoc test for categorical variables or continuous variables. Student’s *t*-test was used to calculate statistically significant differences between the groups in EGFR expression during real-time PCR. Statistical analyses were performed using SPSS package v 23 SAS software version 9.4 (SAS Institute Inc., Cary, NC, USA) [30].

## 3. Results

### 3.1. Measurement of EGFR Levels

**Figure 1:**
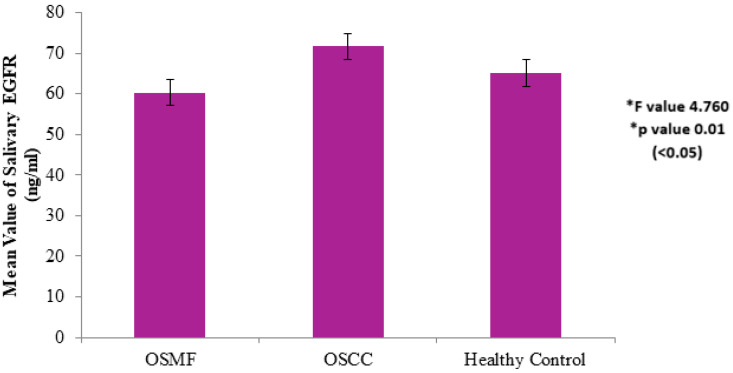
Salivary EGFR levels of healthy controls vs. OSMF vs. OSCC. * Values are expressed as mean ± SD. Statistical significance analyzed by Dunnett’s T3 post-hoc test at *p* < 0.05.

The mean value of salivary EGFR in Group A (OSMF) was found to be 60.32 + 10.2 ng/mL, in Group B (OSCC) was found to be 71.63 + 10.09 ng/mL, and in Group C (healthy controls) was found to be 65.1 + 9.08 ng/mL (Figure 1). The difference was statistically significant for OSCC in comparison with OSMF and healthy controls, with an F value of 4.760 and with *p* value 0.01 (<0.05), using Dunnett’s T3 post-hoc test.

**Figure 2:**
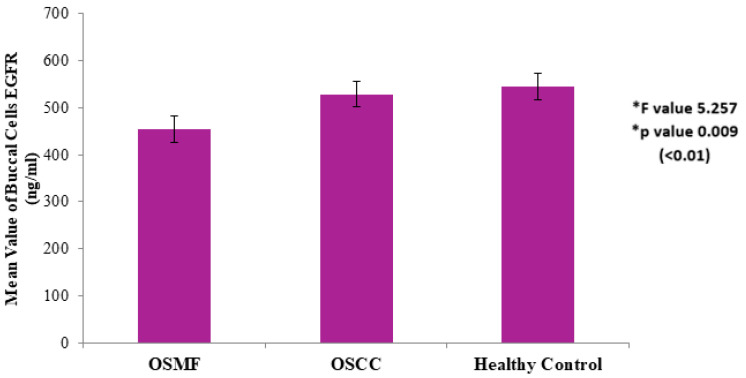
EGFR expression in exfoliated buccal cells in healthy controls vs. OSMF vs. OSCC. * Values are expressed as mean ± SD. Statistical significance analyzed by Dunnett’s T3 post-hoc test at *p* < 0.01.

The mean value of exfoliated buccal cells EGFR concentration in Group A (OSMF) was found to be 454.54 + 89.50 ng/mL, in Group B (OSCC) was found to be 528.79 + 62.25 ng/mL, and in Group C (healthy controls) was found to be 545.47 + 109.72 ng/mL (Figure 2). The difference was found to be statistically significant for OSCC in comparison with OSMF and healthy controls, with an F value of 5.257 and *p* value 0.009 (<0.01), using Dunnett’s T3 post-hoc test.

### 3.2. Immunohistochemistry

**Figure 3:**
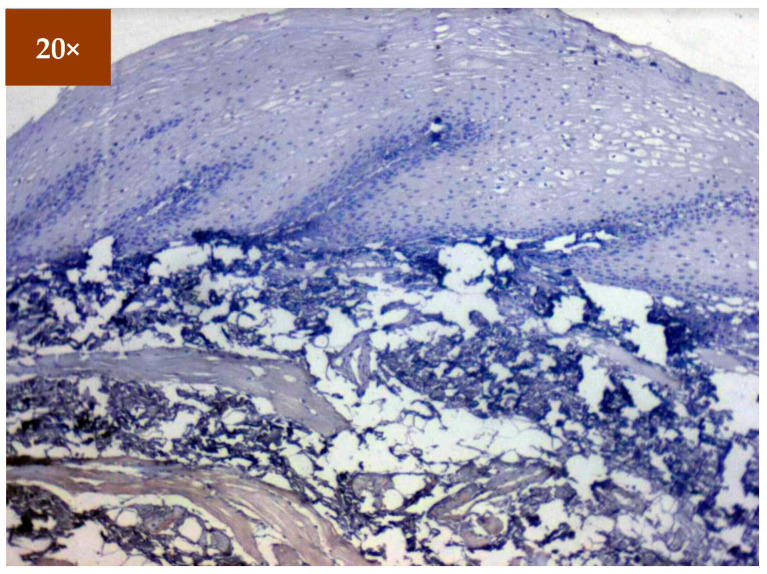
IHC for EGFR in healthy controls (under 20× magnification).

**Figure 4:**
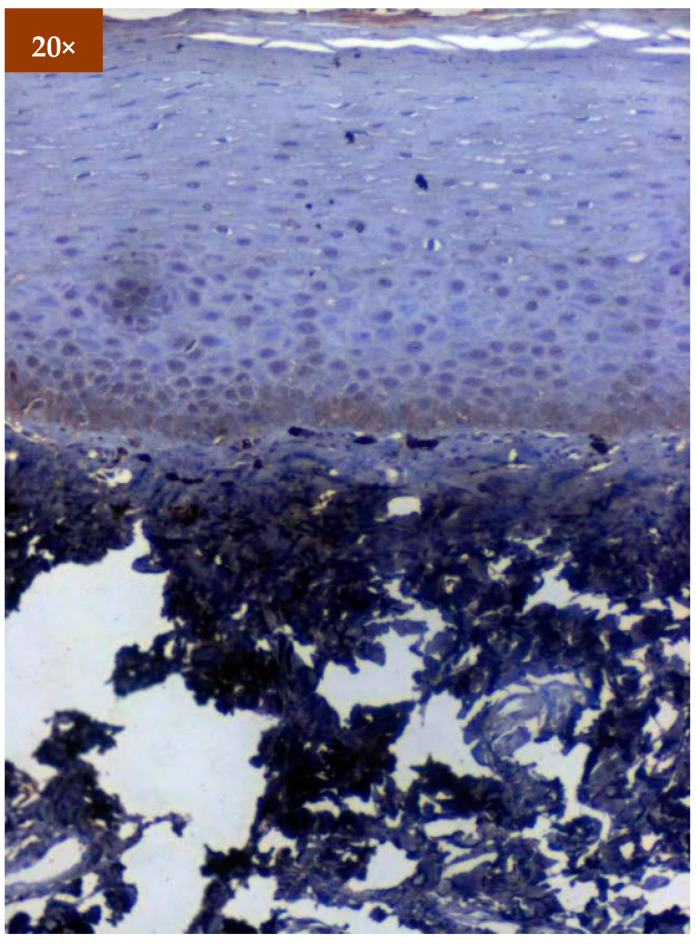
IHC of EGFR in OSMF samples (under 20× magnification).

**Figure 5:**
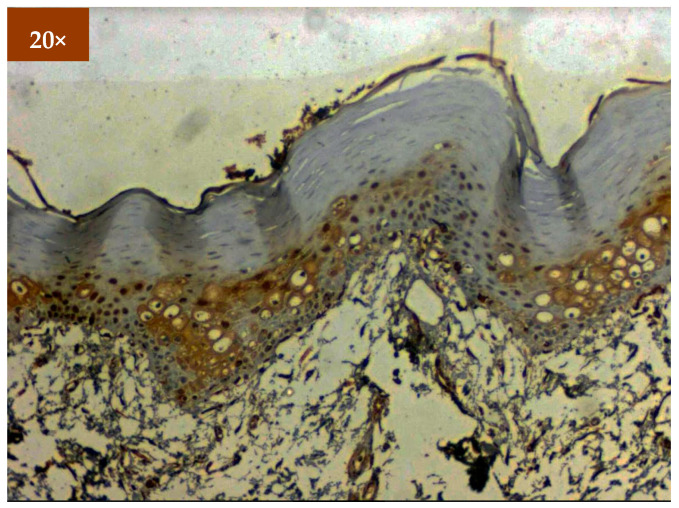
IHC for EGFR in dysplasia (under 20× magnification).

**Figure 6:**
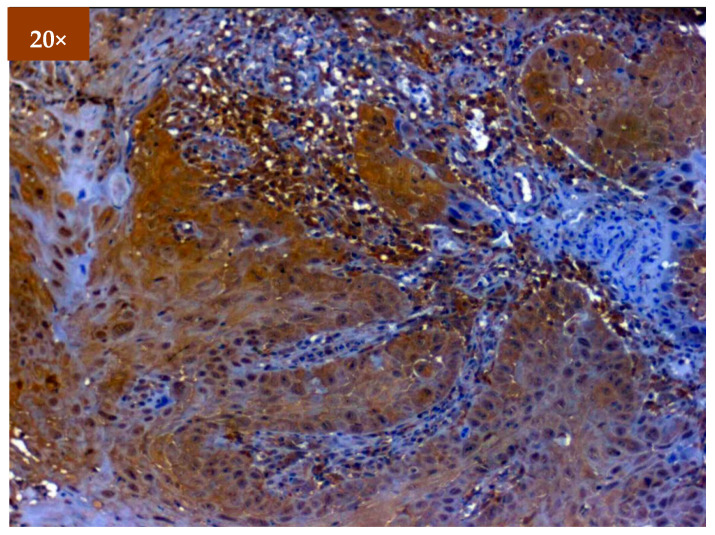
IHC for EGFR in OSCC (under 20× magnification).

Expression of EGFR was studied in 173 patients in different age groups, in which mild positive immunoexpression was found in 23 patients (13.3%), intermediate positive immunoexpression was found in 51 patients (29.5%), strong positive immunoexpression was found in 87 patients (50.3%), and 12 patients (6.9%) tested negative (Table 2). EGFR overexpression was increased in oral cancer and OSMF compared to healthy controls (*p* = 0.000; χ^2^ = 68.620). EGFR overexpression was significantly correlated (*p* = 0.000; χ^2^ = 85.409) with various grades of dysplasia, and well-differentiated (WDSCC) and moderately differentiated squamous cell carcinoma (MDSCC) (Table 3). Figure 3 shows the expression of EGFR in healthy controls showing negative expression. Figure 4 shows the expression of EGFR in OSMF, showing intense cytoplasmic and nuclear positivity. Figure 5 and Figure 6 show the expression of EGFR in dysplasia and OSCC samples, respectively, showing intense cytoplasmic and nuclear positivity. 

### 3.3. Real-Time PCR

**Figure 7:**
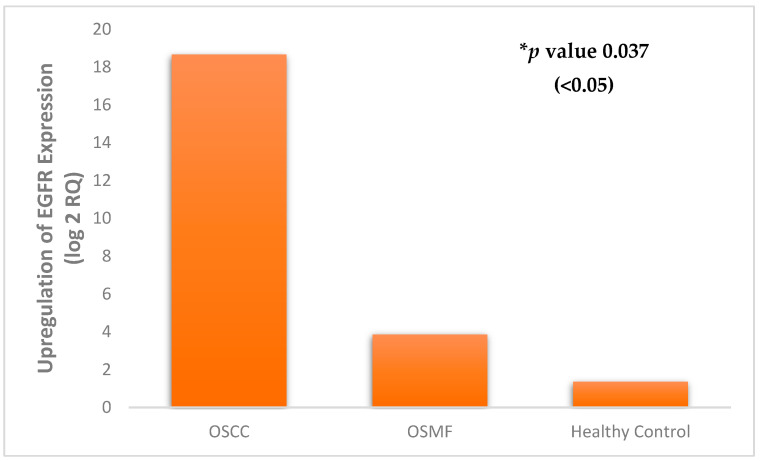
Differential expression of EGFR in healthy controls vs. OSMF vs. OSCC. * Statistical analysis performed using Student’s *t*-test.

The expression of EGFR was studied in tissue samples using real-time PCR for different patient categories (Tables S1 and S2). Based on the obtained results, EGFR exhibited maximum expression in OSCC tissue samples in comparison to OSMF and healthy controls. We found a significant 18-fold upregulation of the EGFR gene in OSCC samples and three-fold upregulation in OSMF samples compared to healthy controls (Figure 7). The EGFR expression was found to be statistically significant using Student’s *t*-test for the studied samples (OSCC vs. OSMF vs. healthy controls), with *p* value 0.037 (<0.05).

## 4. Discussion

The primary function of epidermal growth factor receptor (EGFR) is to maintain homeostasis and epithelial tissue development. Epithelial cancers, including OSCC, have been found to overexpress EGFR and hence it is worth exploring the expression in OPMD [31]. Therefore, early detection of the malignant potential of high-risk OPMD could be evaluated using EGFR, especially using saliva and exfoliated buccal cells as a diagnostic sample, which can prove to be a viable screening tool.

Over the years, numerous techniques, such as IHC, fluorescence in situ hybridization (FISH), and PCR, have been used to identify the overexpression of EGFR, EGFR copy number gains (CNG), and EGFR mutations. However, numerous studies have employed IHC because of its ease and simplicity [32]. Studies evaluating the expression of EGFR in OSCC and leukoplakia have shown a significant correlation in OSCC, when compared to leukoplakia [33,34,35]. The increased expression of EGFR in OPMD such as leukoplakia and OSMF favors EGFR as a valuable diagnostic marker [18]. However, the study mentioned above evaluated the EGFR expression in dysplastic cases of oral leukoplakia and OSMF, with no attempt to compare the grades of dysplasia. In the present study, we have shown significant differences between different grades of dysplasia in OPMD and different histological grades of OSCC. The results of the immunoexpression of EGFR in our study are in accordance with a study performed by Mahendra et al., comparing leukoplakia and OSCC, which showed increased EGFR expression with increasing grades of dysplasia and the highest expression in OSCC [36]. Immunoexpression of EGFR was correlated with the survival rate of oral cancer patients, and the authors have concluded EGFR to be an independent prognostic factor for the assessment of survival rates [37]. With extensive literature support for EGFR using IHC, limited studies are available on saliva and exfoliated buccal cells as a diagnostic sample. Hence, the current study evaluated the EGFR expression in saliva and buccal cells, considering the advantage of patient compliance during screening procedures. The expression of EGFR in saliva was supported valuably by evaluating the immunoexpression in OSCC using IHC and quantification of gene expression using qPCR. In a previous study performed by Bagan et al., evaluating the EGFR copy number using RT-PCR showed significant expression in the advanced stages of oral malignancy compared to OPMD, with higher expression in non-homogenous leukoplakia compared to homogenous leukoplakia [5]. Similar observations were obtained in our study, where EGFR expression was highest in the OSCC group, followed by the OSMF group, compared to healthy controls. The results further validate the role of EGFR in progression to malignancy. EGFR is found to be a transmembrane receptor. However, the extracellular domain of EGFR could be released by proteolytic cleavage and exfoliated from the surfaces of cells; hence, it is found to be detected in other body fluids, mainly saliva [38,39]. In our study, the salivary EGFR levels in OSCC were high, attributed to high EGFR levels in actively dividing tumor cells. This is in accordance with the study performed by Zanotti et al., where the salivary EGFR levels were higher than levels in serum; there was a significant correlation between tumor stage and survival [3]. However, in the study performed by Bernandes et al., salivary EGFR levels were not elevated in OSCC and did not correlate significantly with the clinical pathological parameters [7]. Similarly, in a study comparing salivary EGFR levels in premalignant conditions, OSCC and normal patients did not show a significant association, despite OSCC showing higher salivary EGFR [40].

The present study showed a decrease in salivary EGFR in OSMF compared to OSCC, in accordance with previous studies with OPMD compared to OSCC [35,40]. The reduction in EGFR levels in the saliva of OSMF patients could be attributed to the epithelial atrophy observed in OSMF and the possible pathway of TGFβ-mediated Epithelial to Mesenchymal Transition (EMT) [41]. 

A potential limitation of our study was the smaller sample size and unequal number of samples in different groups for ELISA. However, the attempt to validate the results of saliva using a substantial number of samples in IHC and qPCR sought to overcome this limitation. Thus, the current research could aid in conducting studies with larger samples, with a standardized technique for saliva collection and estimation to obtain reproducible results in confirming the diagnostic utility of salivary EGFR as a biomarker.

## 5. Conclusions

EGFR expression could be considered a promising diagnostic marker and its evaluation in saliva and exfoliated buccal cells using ELISA could serve as a valuable screening diagnostic tool for OPMD. The validation studies using IHC have shown increased immunoexpression of EGFR in OSCC compared to OPMD. Furthermore, gene expression studies performed using RT-PCR validate the upregulation of EGFR in OSCC in comparison to OSMF and healthy controls. Further studies with larger samples of OSMF and OSCC patients will shed light on the reliability of salivary EGFR as a diagnostic marker in identifying the possible malignant risk.

## Figures and Tables

**Table 1:** EGFR primer sequences.

S. No.	Gene Name	Forward Sequence	Reverse Sequence
1.	EGFR	5’-AGGCACGAGTAACAAGCTCAC-3’	5’-ATGAGGACATAACCAGCCACC-3’

**Table 2:** Clinical histopathological features in EGFR-positive groups in healthy controls and OSMF and OSCC patients.

Type	Negative	Mild EGFR Positive	Intermediate EGFR Positive	Strong EGFR Positive	Total (n = 173)
Healthy controls	5 (41.7%)	2(8.7%)	4 (7.9%)	0 (0%)	11
OPMD with dysplasia	5 (41.7%)	16 (69.6%)	22 (43.1%)	13 (14.9%)	56
OSCC	2 (16.6%)	5 (21.7%)	25 (49%)	74 (85.1%)	106
Total	12 (6.9%)	23 (13.3%)	51 (29.5%)	87 (50.3%)	173
				*p* = 0.000; χ^2^ = 68.620	

**Table 3:** Clinical histopathological features in EGFR-positive groups in dysplasia and cancer patients.

Type	Negative	Mild EGFR Positive	Intermediate EGFR Positive	Strong EGFR Positive	Total (n = 173)
Healthy Controls	5 (41.7%)	2 (8.7%)	4 (7.8%)	0 (0%)	11
Mild dysplasia	2 (16.7%)	9 (39.1%)	11 (21.6%)	3 (3.4%)	25
Moderate dysplasia	2 (16.7%)	7 (30.4%)	8 (15.7%)	3 (3.4%)	20
Severe dysplasia	1 (8.3%)	0 (0%)	3 (5.9%)	7 (8%)	11
WDSCC	1 (8.3%)	3 (13%)	10 (19.6%)	45 (51.7%)	59
MDSCC	1 (8.3%)	2 (8.7%)	15 (29.4%)	29 (33.3%)	47
				*p* = 0.000; χ^2^ = 85.409	

## Data Availability

The study did not report any additional data.

## Supplementary Materials

The following supporting information can be downloaded at: https://www.mdpi.com/article/10.3390/diagnostics12081935/s1. Table S1: Expression of EGFR in OSCC samples; Table S2: Expression of EGFR in OSMF samples.
