# Supplementary material for: Identification of EGFR as a Biomarker in Saliva and Buccal Cells from Oral Submucous Fibrosis Patients—A Baseline Study
Source: Diagnostics (Basel). 2022 Aug 11;12(8):1935. doi: 10.3390/diagnostics12081935 (PMC9406318; doi:10.3390/diagnostics12081935)
Supplement: Supplementary file 1 [file diagnostics-12-01935-s001.zip › diagnostics-1851977-supplementary.pdf]

Table S1. Expression of EGFR in OSCC samples

Sample ID	Number	EGFR	GAPDH	dCt	ddCt	2^-ddCt
	Control/Normal	23.56	16.76	6.8		
T1	429T	22.82	18.14	4.68	-0.95	1.931872658
T2	496T	21.97	18.15	3.82	-3.07	8.397733469
T3	438T	19.05	16.18	2.87	-3.93	15.24220797
T4	503T	19.33	14.75	4.58	-4.88	29.44600482
T5	449T	17.93	16.05	1.88	-3.17	9.000467878
T6	650T	17.05	14.01	3.04	-5.87	58.48521281
T7	405T	17.99	13.33	4.66	-4.71	26.17286587
T8	663T	17.05	13.87	3.18	-3.09	8.51496146
T9	518T	18.23	12.68	5.55	-4.57	23.75237713
T10	423T	21.17	14.86	6.31	-2.2	4.59479342
T11	445T	17.26	12.67	4.59	-1.44	2.713208655
T12	270T	19.94	16.31	3.63	-3.16	8.938297105
T13	117T	26.25	17.57	8.68	-4.12	17.3877578
T14	431T	17.1	12.28	4.82	0.93	0.524858342
T15	245T	32.01	16.74	15.27	-2.93	7.621103984
T16	381T	20.4	16.45	3.95	7.52	0.005448217
T17	604T	25.34	20.06	5.28	-3.8	13.92880901
T18	440T	19.15	16.18	2.97	-2.47	5.540437872
T19	449T	20.2	13.85	6.35	-4.78	27.47409397
T20	154T	14.79	12.77	2.02	-1.4	2.639015822
T21	217T	16.23	14.69	1.54	-5.73	53.07645093
T22	369T	16.02	13.84	2.18	-6.21	74.02804377
T23	335T	19.09	13.47	5.62	-5.57	47.50475426
T24	367T	17.98	14.17	3.81	-2.13	4.377174805
T25	277T	16.12	13.12	3	-3.94	15.34822591

Table S2. Expression of EGFR in OSMF samples

Sample ID	Name	EGFR	GAPDH	dCt	ddCt	2^-ddCt
	Control/Normal	**23.56**	**16.76**	6.8	-0.95	
O1	Confidential	24.86	13.46	11.4	3.65	0.079660039
O3		25.38	15.04	10.34	2.59	0.166085727
O4		25.85	18.89	6.96	-0.79	1.729074463
O8		24.54	15.09	9.45	1.7	0.307786103
O9		24.99	18.1	6.89	-0.86	1.815038311
O10		29.39	21.93	7.46	-0.29	1.222640278
O12		20.65	17.09	3.56	-4.19	18.25221945
O14		23.63	14.32	9.31	1.56	0.339151082
O15		16.42	12.11	4.31	-3.44	10.85283462
